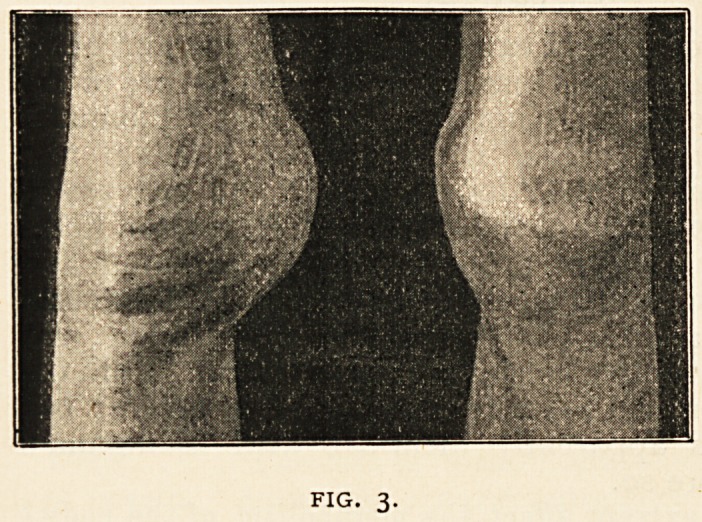# A Case of Hæmophilia with Joint Lesions

**Published:** 1897-09

**Authors:** J. E. Shaw

**Affiliations:** Professor of Medicine, University College, Bristol; Physician to the Bristol Royal Infirmary


### A CASE OF HÆMOPHILIA WITH JOINT-LESIONS.[1]

BY

J. E. Shaw, M.B. Ed.,

*Professor of Medicine, University College, Bristol;*
*Physician to the Bristol Royal Infirmary.*

A short account of the subject of this notice was published by the late Mr. Greig Smith.[2] At the risk of some repetition, however, an outline of the clinical history must be given now to explain the full meaning of the illustrations.

Alfred P., aged 30, single, a relief-stamper by trade, was admitted to the Bristol Royal Infirmary on March 16th, 1897, complaining of passing blood in his urine.

Family History.—Two brothers died in infancy from hemorrhage, aged fifteen and seventeen months respectively—in one case from hemorrhage from the mouth, and in the other from the opening by incision of a small abscess of the scalp. Two other brothers and three sisters

[1] Read at the May meeting of the Bristol Medico-Chirurgical Society.

[2] *Bristol M.-Chir. J.*, 1884, ii. 264.

have grown up without manifesting the hemorrhagic diathesis. A maternal uncle is thought to have died of bleeding, but this is not absolutely certain. There is no other fact in the ascertainable family history pertinent to the question of hereditary liability to hemorrhage.

Previous History.—Patient was born in London, and at seventeen months of age was admitted to St. Thomas's Hospital with swelling of the left knee. He has been told that the swelling burst, and that the wound was kept open for a month; he remained in hospital on this occasion six months. Within two months of his discharge he was re-admitted on account of the diseased left knee. At five years of age he was an inmate of the Bristol Hospital for Sick Children, with his left knee bad as before. At seven years of age he was in the Bristol General Hospital on account of the condition of the same joint. At eleven years of age patient was struck with a stone on the inner side of the right knee; the joint became very much swollen, and he was brought to the Bristol Royal Infirmary under the care of the late Mr. Tibbits, and remained there on this occasion four months. The right knee and elbow were aspirated, and blood was withdrawn from both joints. Fig. 1 shows him as he was at this time, before aspiration of the joints was performed. There is hæmarthrosis of right knee and elbow, subsequently relieved by the aspiration; slight enlargement of the left knee is also visible. Shortly after this he suffered from hæmatemesis for two months. At thirteen years of age he suffered severely from epistaxis. At seventeen years of age he was again in the Bristol Infirmary under Mr. Greig Smith, suffering from uncontrollable hemorrhage from a wound of the left thumb. This is the occasion when Mr. Greig Smith described his case as above-mentioned. He remained an in-patient for three weeks, and in two months more he was re-admitted for hemorrhage from the gums. Again at the ages of eighteen and twenty years he was in the Bristol Infirmary under Mr. Greig Smith for bleeding from the gums; at the latter age he had an attack of small-pox, which was not hemorrhagic at all. At twenty-one

FIG. 1.

years of age he is said to have had an attack of rheumatic fever. Two years ago, when aged twenty-eight, patient had a tooth extracted, followed by hemorrhage for a week. He states that hemorrhage from the gums is preceded for one or more days by twitching of the eyelids. Since early childhood he has been subject to temporary pains of an acute nature, affecting the larger joints of the limbs, accompanied by swelling: this swelling is always preceded by a "jumping" sensation and feeling of heat.

History of Present Illness. — Six days ago patient noticed that his urine was dark brownish-red. There has been neither pain nor difficulty in micturition during this period, although the colour has been so abnormal. The frequency of micturition and the amount of urine passed have not deviated from the normal.

Condition on Admission.—Thin and rather cadaverous in appearance, complexion sallow, face marked with scars of small-pox. Height, 5 ft. 8 ins.; weight, 8 st. 9 lbs. Temperature subnormal. *Circulatory and respiratory systems:* nothing specially abnormal. *Digestive system*: lips pale, but not excessively so, gums spongy and liable to bleed, teeth very carious, breath fetid. *Hæmopoietic system:* spleen, thyroid and lymphatic glands all normal. Blood contained 55 per cent. of hæmoglobin, corpuscles 4,000,000 per c.m., leucocytes slightly in excess of the normal. *Nervous system:* knee-jerk scarcely present. *Urinary system:* urine of deep brownish-red colour was found to contain much hæmoglobin and albumen; microscopically the deposit was found to be composed of blood corpuscles and urates, there were no casts of any kind, nor epithelium. *Arthritic system:* both elbows and both knees are larger than normal; the styloid process of each ulna is enlarged, as also to some degree are the lower ends of each tibia. The upper radio-ulnar joint of each arm is larger than normal, from enlargement of the head of the radius, which in rotation of the fore-arm seems to move eccentrically. There is no free fluid in these joints, the enlargement being bony. The elbow-joints can be flexed to the normal degree, but extension is impossible beyond an angle of about 130°. The knees are always flexed, the left the more so; the left knee can be moved through an angle of about 35° only, the right 75°. Movements in all these joints gives rise to a coarse grating or crunching sound, and is sometimes painful. At the edges of the articular surfaces of the bony structures forming the knee-joints marked "lipping" can be felt on palpation; the phalangeal, metacarpo-phalangeal and metatarso-phalangeal joints are not affected, nor apparently the hip and shoulder joints.

Subsequent History.—In the days following that of admission, patient passed upon one or two isolated occasions urine free from blood, which was also non-albuminous, otherwise the hæmaturia persisted in variable degree of severity. Upon several occasions he had attacks of severe "colic" associated with the left kidney, requiring even hypodermic administration of morphia for the relief of the pain; these attacks were followed by the passing of blood-clots in the urine. Many examinations of the blood were made during the patient's stay in the Bristol Infirmary; the hæmoglobin fell to 28 per cent., and the corpuscles to 3,500,000 on April 18th. In many films examined, both stained and unstained, no special nor permanent abnormality was observed: the leucocytes seemed sometimes in excess of normal, and sometimes not so, and towards the end of his stay the multinuclear leucocytes were partly replaced by lymphocytes. No retinal hemorrhages occurred, and the gums only bled occasionally. The urine became finally free from blood on April 20th, nearly five weeks after admission. In addition to the permanently enlarged condition of certain of the joints of his extremities, he suffered from frequent attacks of acute effusion around and *perhaps* slightly into these same joints; these attacks were preceded by definite prodromata : several hours before the joint-lesion began, there was always a feeling of "pins and needles" in the fingers or toes of the same limb, and a feeling of heat in the joint. The next morning there would be considerable swelling superficial to the ligaments of the joint and beneath the true skin, and one or more spots about the area of a shilling or less would be extremely tender on pressure, at the same time the joint would be extremely painful on being flexed or extended. It was doubtful if any effusion took place into the synovial sac itself on these occasions; if there were any such, it was extremely slight. These attacks were not induced by use, exertion, or trauma, but occurred without obvious exciting cause. The patient could always prophesy infallibly when any joint was going to be thus affected, from the occurrence of the sensory phenomena about twelve hours before the swelling. The condition usually passed off in two days. Some ecchymosis followed each puncture by the hypodermic syringe; but in pricking the finger for blood examination some difficulty was encountered in obtaining blood, but none in checking hemorrhage from these punctures.

In the way of *treatment* he was given calcium chloride for the first three weeks in doses of eighty grains per diem; to this was added on April 1st three tabloids of thymus gland a day; on April 7th the calcium chloride was replaced by sulphuric acid and quinine, and this again on April 15th by oleum terebinthinæ in doses of half a drachm a day. After the cessation of the hæmaturia he was ordered iron and quinine only. Operative procedure was not seriously considered, as the hemorrhage never put his life in actual danger, and only an urgent necessity would have justified cutting down upon the kidney.

The case is one of considerable interest. Firstly, the patient has lived beyond the age to which hæmophiliacs usually survive, partly, no doubt, from the skilful treatment he has so often received in hospitals since infancy. Should his life be prolonged, he will probably lose his hemorrhagic tendency.

Secondly, the evolution of the present condition of his affected joints has been watched from infancy also. At St. Thomas's Hospital he was supposed to have "white swelling" of the knee. In 1878 the right knee and elbow were aspirated, and free blood withdrawn from each joint, and the case then recognised as one of hæmophilia. Now he appears to get very little or no effusion into the joints, but instead the curious peri-articular lesions before described, preceded by sensory phenomena, while hemorrhage from the gums is preceded by motor phenomena also. The affected joints are found by examination and shown by skiagraphic delineation to be in a condition similar to that of rheumatoid arthritis. The articular ends of the bones are much enlarged, the cartilages extensively absorbed, and very distinct

"lipping" exists at the edges of the articular surfaces. A skiagram (Fig. 2) of the right elbow-joint is here given. It

FIG. 2.

shows great enlargement of the head of the radius, some "lipping" at the edge of the sigmoid fossa of the ulna, and thinning of the articular cartilages. Even in recent literature the joints in this disease are imperfectly described, and are said to resemble, more or less closely, tubercular joints. Positive error upon this ground is sometimes made. J. E. Summers, Jun., reports[3] a case in which an enlarged ankylosed knee, occurring in a boy aged ten years, was diagnosed as "quiescent tubercular arthritis." The joint was resected, and the patient died in twenty-four hours from uncontrollable hemorrhage, the operator discovering too late that he was treating a hæmophiliac. Bowlby,[4] in a paper entitled "Some Cases of Joint-Disease in Bleeders," gives a true description of the nature of the lesion. Skiagrams were taken of the knees also in this case; but owing to the inability of the patient to extend his knees, the results were not very clear, and the prints therefore are not here reproduced, but great enlargement of the condyles and "lipping" were apparent. A photograph (Fig. 3) from the front shows great enlargement of the articular ends of the femora and tibiæ.

[3] *Med. Rec.*, 1896, xlix. 336. [4] *St. Barth. Hosp. Rep.*, 1890, xxvi. 77.

Thirdly, it is to be noticed that unlike rheumatoid arthritis, the fingers and toes completely escape implication.

Fourthly, medicinal treatment probably had little effect. Calcium chloride failed to exert any styptic action, and nuclein administered in the form of thymus gland did not apparently act as a stimulant to hæmopoiesis, or even leucocytosis. Treatment can hardly be otherwise than unsatisfactory where the pathology is unknown.

FIG. 3.

Fifthly, the nerve symptoms preceding the acute attacks in the joints would suggest that these lesions are a form of spinal arthropathy.

Sixthly, the hereditary element in this case appears to be defective; but persons in this class of life seldom know much of the medical history of their ascendants, and this case may possibly be as truly hereditary as others from well-known "bleeder" families.

Lastly, Senator's oft-quoted case would have justified nephrectomy, had life been placed in actual danger from the hemorrhage.

---

After the narration of Dr. Shaw's case, Dr. G. PARKER said it would be interesting to know if in hæmophilia there was an absence of contraction of the clot and its subsequent concavity. It was said by Bensaude that this does not occur in purpura hæmorrhagica, though it does in normal blood and in rheumatic purpura.

## Figures and Tables

**Fig. 1. f1:**
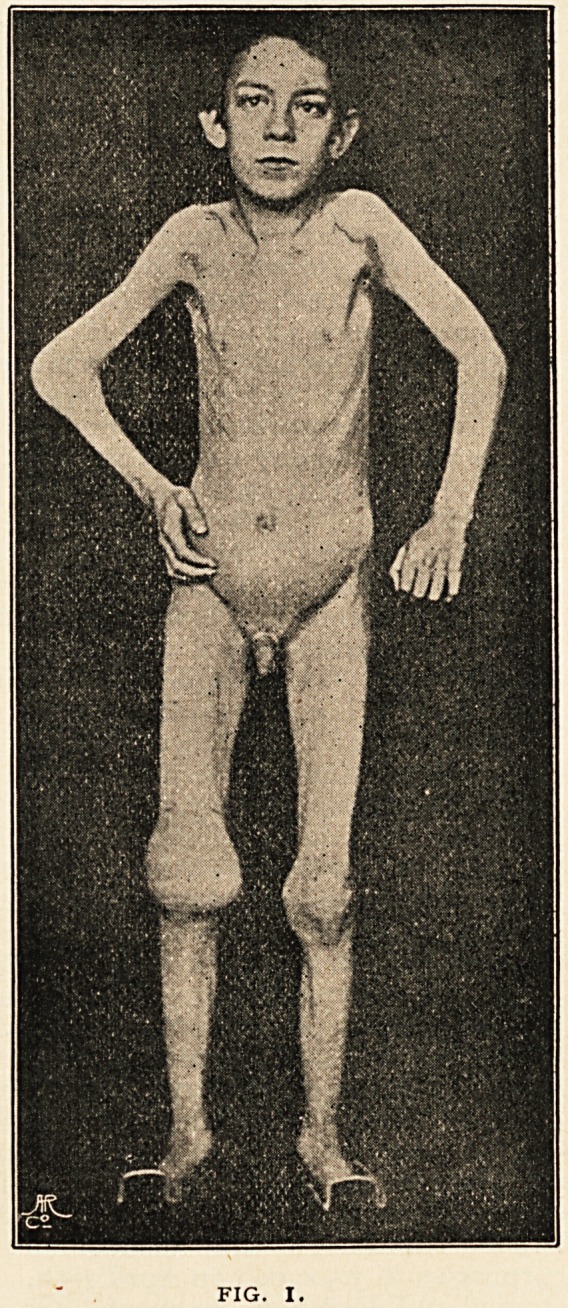


**Fig. 2. f2:**
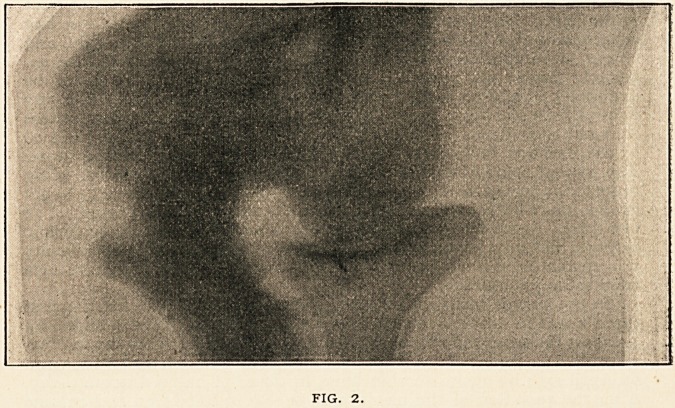


**Fig. 3. f3:**